# Preclinical IV busulfan dose-finding study to induce reversible myeloablation in a non-human primate model

**DOI:** 10.1371/journal.pone.0206980

**Published:** 2018-11-29

**Authors:** Nadim Mahmud, Amit Khanal, Simona Taioli, Emre Koca, Sujata Gaitonde, Benjamin Petro, Karen Sweiss, Lisa Halliday, Xinhe Wang, Pritesh Patel, Damiano Rondelli

**Affiliations:** 1 Division of Hematology/Oncology, Department of Medicine, University of Illinois college of Medicine, Chicago, Illinois, United States of America; 2 University of Illinois Cancer Center, University of Illinois Hospital & Health Sciences System, Chicago, Illinois, United States of America; 3 Department of Pathology, University of Illinois Hospital & Health Sciences System, Chicago, Illinois, United States of America; 4 Department of Pharmacy Practice, College of Pharmacy, University of Illinois Hospital & Health Sciences System, Chicago, Illinois, United States of America; 5 Biologic Resources Laboratory, University of Illinois College of Medicine, Chicago, Illinois, United States of America; 6 Division of Epidemiology and Biostatistics, School of Public Health, University of Illinois at Chicago, Chicago, IL, United States of America; St. Vincent's Institute, AUSTRALIA

## Abstract

In this study we utilized a large animal model to identify a dose of intravenous busulfan that can cause reversible myelosuppression. Nine baboons (*Papio anubis*) were treated with IV busulfan at 6.4 (Group A), 8 (Group B), or 9.6 mg/kg (Group C). Peripheral blood counts were measured up to 90 days after treatment and serial bone marrow samples were obtained to analyze CD34+ cell content and colony forming units. Overall, the highest grade of peripheral blood cytopenia was observed 15 days after treatment in all three groups (n = 3/group). In particular, we observed a notable reduction of neutrophil and platelet counts in the blood and the number of marrow CD34+ cells and colony forming units. In contrast, the effect of busulfan on hemoglobin levels was mild. Baboons who received the highest dose of busulfan showed only a 25–35% recovery of marrow CD34+ cells and colony forming units after 90 days of busulfan administration. However, all three groups of animals showed a full recovery of peripheral blood counts and normal marrow cellularity and tri-lineage hematopoiesis after treatment. Notably, all three doses of busulfan were tolerated well without significant extra-medullary toxicity. These results validate the hierarchy of blood cells likely targeted by busulfan, and based on these findings, clinical trials using myelotoxic but not myeloablative doses of intravenous busulfan will be designed for patients with myeloid malignancies.

## Introduction

Busulfan is an alkylating agent with myelosuppressive effects which exhibits a prominent effect on subsets of hematopoietic progenitor cells defined by lineage commitment and differentiation stage as well as non-cycling cells [1]. Busulfan was first used at low doses in the treatment of chronic myelogenous leukemia (CML) and other chronic myeloproliferative neoplasms in the 1950s [2]. Because patients treated with long-term, low-dose busulfan eventually developed marked pancytopenia with marrow hypocellularity it was hypothesized that toxicity of busulfan against hematopoietic stem cells (HSC) occurs even in the absence of profound myelosuppression [3]. In addition, previous studies in a feline model suggested damage to the HSC reserve years after busulfan exposure [4], as well as a significant toxic effect of low doses of busulfan on HSC and progenitor cell clonal dynamics [5]. In contrast, high doses of oral busulfan elicit a potent and rapid myelotoxicity and have been used for over 40 years in the conditioning regimen of myeloablative hematopoietic stem cell transplantation (HSCT), especially for patients with acute or chronic malignancies [6–12]. However, despite the fact that busulfan is the chemotherapy of choice in preparative regimens for HSCT in myeloid malignancies, neither busulfan nor any other alkylating agents is usually included in first line or salvage protocols for patients with acute myeloid leukemia (AML). One of the reasons could be the lack of an experimental model to test therapeutic doses of busulfan that would not require a stem cell rescue to recover normal hematopoiesis.

Because of a high biological and developmental similarity between non-human primates (NHP) and humans, including comparable immune and hematopoietic systems, NHP such as the *Papio Anubis* baboon model represent an excellent resource to conduct preclinical studies investigating responses to hematologic stress such as chemotherapy. Here we utilized this NHP model to test different non-myeloablative doses of intravenous (IV) busulfan with the aim of inducing a reversible neutropenia, similar to what is observed in patients with AML receiving standard chemotherapy. To better characterize the effect of IV busulfan we also analyzed the bone marrow (BM) of these animals for marrow cellularity and morphologic changes as well as changes in CD34+ cell numbers and clonogenic potential of BM cells. As well, the number and the *in vitro* clonogenic function of hematopoietic stem progenitor cells (HSPCs) at different time points after busulfan administration were examined. The results shown in this study will prompt the design of new clinical trials with IV busulfan for patients with AML resistant to standard treatment with anthracyclines.

## Materials and methods

### Animals

Adult healthy female baboons (*Papio anubis*) were used for the study and their physical characteristics are outlined in Table 1. Due to the lack of an appropriate number of male baboons in our facility the studies are limited to female only. The baboons were housed in conditions in conformance with the Association for the Assessment and Accreditation of Laboratory Animal Care and the protocol was approved by the Office of the Animal Care and Institutional Biosafety (OACIB), Illinois Institutional Animal Care and Use Committee (IACUC), University of Illinois at Chicago (Protocol number: ACC 11–203). The study animal welfare, supportive care for specific treatment, housing, feeding, etc. and other details are provided below.

**Table 1:** Baboon characteristics.

Group	Baboon Number	Age (Years)	Sex	Body weight (kg)
**Group A**	**PA 7518**	12	F	26.1
	**PA 7520**	17	F	18.5
	**PA 7540**	12	F	22.1
**Group B**	**PA 6537**	22*	F	17.5
	**PA 6683**	21*	F	18.1
	**PA 7519**	12	F	27
**Group C**	**PA7527**	16	F	18.6
	**PA7521**^**¶**^	18	F	20.1
	**PA 7528**	14	F	21.8
	**PA 7533**	15	F	18.2

*arrived in animal colony as adult estimated age at arrival ~6 years

^¶^Animal deceased 16 days’ post busulfan

#### Supportive care for animals

Supportive care for the study baboon were based on clinical need following the approved protocol. Naxcel (ceftiofur, 2.2 mg/kg, SQ or IM, SID) was administered prophylactically against bacterial infections if white blood cell count drops below 2,000/μl or absolute neutrophil count below 500/μl). We did not anticipate requiring blood transfusions since permanent myeloablation was not our objective in this study and the highest dose of busulfan proposed is profound but only transient myeloablation was expected. The proposed non-myeloablative dose of busulfan was not likely to require blood transfusions. In an unlikely event ABO matched irradiated blood would be available for transfusion.

The animals were monitored daily for pre-established endpoint criteria for the first 2 weeks and euthanized if they become recumbent, nonresponsive, lose 20% of their baseline body weight, or develop respiratory distress. The animals were monitored for a total period of 90 days. If the blood counts both WBC and platelets returns to pretreatment levels the animals will be considered completely recovered.

The adverse effects of busulfan include profound and permanent myelosuppression (complete) which is not expected in our current studies since a non-myeloablative dose will be used. In addition, rare cases of neurotoxicity including seizures have been noted in humans. In addition, nausea, vomiting, diarrhea, hepatotoxicity (increased transaminases) or mild to moderate dyspnea has been noted. In addition, occasional cases of skin rash and pruritis can also occur. Symptomatic measures would be taken to minimize pain and discomfort as needed based on veterinary discretion. Prophylactic benzodiazepine (Lorazepam 0.02 to 0.05 mg/kg IV) would be administered 30 minutes before administering busulfan to prevent seizures as has been utilized for humans [4–6]. In addition, Zofran 0.1 to 0.2 mg/kg IM would be used as a prophylactic antiemetic. Use of phenytoin would be avoided as it has been noted to enhance drug clearance of busulfan diminishing drug bioavailability. Before starting therapy as well as following the administration of IV busulfan all animals would undergo tests for blood chemistry, complete blood counts (CBC), hepatic and renal function tests including chest x-ray to assess non-hematologic toxicity. The animals were assessed daily for health status as well as weekly physical exams by a veterinarian.

#### Peripheral blood and bone marrow aspiration

Each time blood was drawn, 4 to 5 ml of blood was collected from a peripheral vein. Peripheral blood (PB) was drawn prior to busulfan administration once, and following busulfan administration PB was collected a minimum of twice a week for 4 weeks or until the blood WBC and platelet counts return to pre-treatment levels, whichever occurred first. Once blood counts were normalized, PB samples were drawn for CBC and differential counts every 2 to 4 weeks until day 90 after busulfan therapy. At one time only a maximum of 20 to 30 ml of BM, much lower than 15% of their circulating blood volume, was drawn.

#### Housing and cage size

Study animals were housed in a dedicated housing facility which is our accredited animal facility (Biologic Resource Laboratory). Animals were housed in individual cage and housed in a room with other animals. During study period the animal was expected to experience partial myeloablation so was housed in a separate room with a healthy companion animal in a separate cage.

Cage size: Colony housing for female baboons was 16.2 sq. ft. floor space and 40” height. The tether cages provided 8.1 sq. ft. floor space and 40” height.

#### Feeding

Baboons were fed a commercial Old World primate diet that provided 15% protein once per day in the morning. In the afternoon, they received a food supplement consisting of fresh produce or a seed/nut foraging mix.

#### Environmental enrichment

Adult baboons were provided social contact to conspecifics through grooming panels. They also received food enrichment that was hidden in various substrates and disposable containers to provide occupational enrichment. Visual and auditory enrichment were provided through music and animated movies. Perches were present in cages for structural enrichment.

#### Euthanasia of study animals

Animals were euthanized following approved protocol. For baboon, sodium pentobarbital 1 ml/10 lbs body weight IV is utilized.

### Test dose and PK studies

The IV busulfan test dose was 0.8 mg/kg using actual body weight as previously described [13]. To conduct pharmacokinetics (PK) studies busulfan was administered as IV infusion (rate: 0.8 mg/kg) over 30 minutes under ketamine sedation. Blood specimens (2–3 ml) were drawn from a peripheral vein in the arm opposite to the IV line where busulfan was infused. Six serial blood samples were collected at the following times: immediately before busulfan administration, immediately after the completion of busulfan infusion, 20 minutes, 35 minutes, 50 minutes, 4 hours, and 6 hours from the start of busulfan infusion. The blood samples were placed on wet ice immediately and centrifuged for 10 minutes at 3000–3200 rpm. The plasma was collected in plastic tubes with screw caps, frozen at– 20^o^ C and shipped out on dry ice to the Seattle Cancer Care busulfan monitoring pharmacokinetics laboratory (Seattle, WA).

### Treatment design

**Fig 1:**
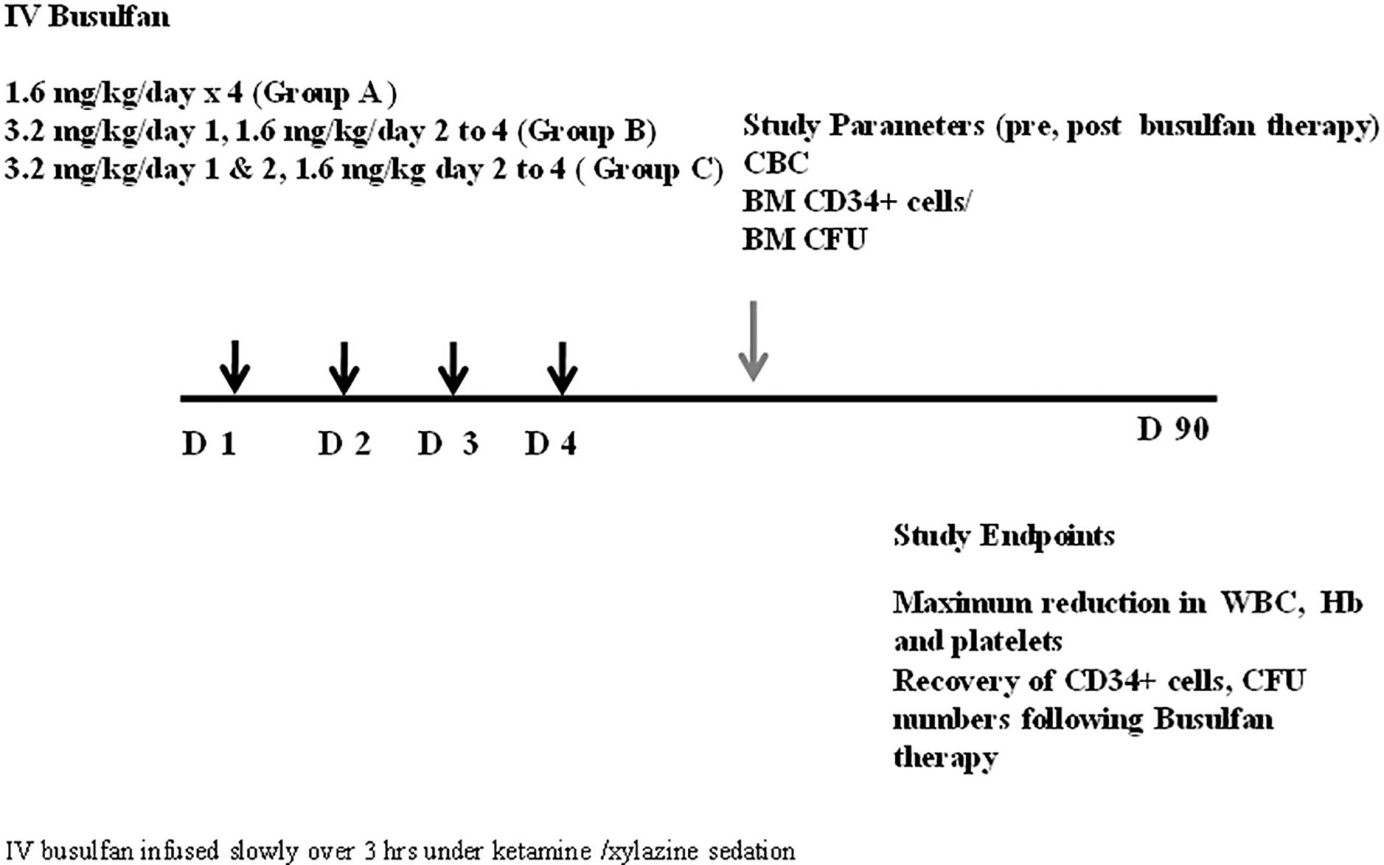
Study schema for IV busulfan to induce reversible myelosuppression without stem cell rescue in a pre-clinical baboon model.

As shown in Fig 1, the animals were divided into three groups (A, B and C) based on the IV busulfan dose they received (n = 3/group). Group A received a total dose of 6.4 mg/kg of IV busulfan, given as 1.6 mg/kg per day for four days. Group B received a total dose of 8 mg/kg, given as 3.2 mg/kg on day 1 and 1.6 mg/kg from day 2 to 4. Group C received a total dose of 9.6 mg/kg, given as 3.2 mg/kg on days 1 and 2, and 1.6 mg/kg on days 3 and 4. The non-myeloablative dose of busulfan was guided by pharmacokinetic studies performed in humans by our group previously [13] and that were confirmed in our first baboon on study (not shown). IV busulfan was obtained from Otsuka Pharmaceutical Development and Commercialization, Inc. (Princeton, NJ). IV busulfan for transient myeloablation was administered over 3 hours unlike studies for PK studies (30 minutes) due to the volume difference.

As part of monitoring procedures, the body weights of the animals were measured prior to the start of busulfan treatment and weekly until day 90 to determine extra hematopoietic toxicity.

### Peripheral blood and bone marrow

Peripheral blood (PB) was obtained following standard procedure by venipuncture under ketamine sedation and analyzed for complete blood count (CBC). BM aspiration and core biopsy was also performed under ketamine and xylazine sedation as described previously [14]. For morphological examination of three lineages of blood following busulfan administation serial BM biopsy samples were stained with haematoxylin and eosin and examined until day 90.

CBCs were done at the Biologic Resources Laboratory using an ADVIA 120 hematology analyzer (Siemens Healthcare Diagnostics, New York, NY). Differential counts were performed manually using a light microscope (Olympus, Waltham, MA) from Giemsa stained PB smears.

The collection and separation of BM and PB cells were performed as described previously [15,16]. Heparinized BM and PB samples were diluted with phosphate-buffered saline (Mediatech, Manassas, MA) and light density mononuclear cells (MNC) fraction were obtained by density gradient centrifugation over 60% percoll (GE Life Sciences, Marlborough, MA) as described previously [16].

### Flow cytometry

BM MNCs were stained using mouse IgM anti-CD34 (12.8; a gift of Dr. Robert G. Andrews, Fred Hutchinson Cancer Research Center, Seattle, WA) or matched isotype control (Southern Biotechnology, Birmingham, AL), and FITC conjugated goat anti mouse IgM secondary antibody (Southern Biotechnology). In addition, FITC conjugated mouse anti CD3 monclonal antibody reactive against baboon T-lymphocytes and matched isotype control antibody conjugated with FITC (Becton Dickinson, San Diego, CA) was used to stain BM CD3+ T lymphocytes. Cells were analyzed on a FACSCalibur flow cytometer (Becton Dickinson, San Jose, CA) and at least 10,000 live cells were acquired.

### CFU assays

Colony forming cells were assayed by plating BM MNC in semisolid 1.1% methyl cellulose based media containing 30% FBS, 50 μM 2-mercaptoethanol, and a cocktail of human recombinant cytokines consisting of 100 ng/mL stem cell factor, 100 ng/mL Fms-like tyrosine kinase-3 (Flt3) ligand, 50 ng/mL interleukin3, 50 ng/mL IL-6, 50 ng/mL granulocyte-monocyte colony-stimulating factor, and 5 U/mL erythropoietin (Stem Cell Technologies, Vancouver, BC, Canada) as described previously [15,16]. The colonies were counted after 14 days following standard criteria as described previously [15,16].

### Data analysis and statistics

Data are expressed as mean ± standard error (SE) of three animals in each individual group. For statistical analyses, paired (within same group of baboons) or unpaired (inter groups) Student’s t-test was used as deemed appropriate. The relationship between BM colony forming unit (CFU) recovery and the following measures were examined using Pearson correlation coefficient: CD34+ cell content recovery, recovery of absolute neutrophil count (ANC) in PB, and PB white blood cell (WBC) count.

## Results

### Effects on peripheral blood counts

**Fig 2:**
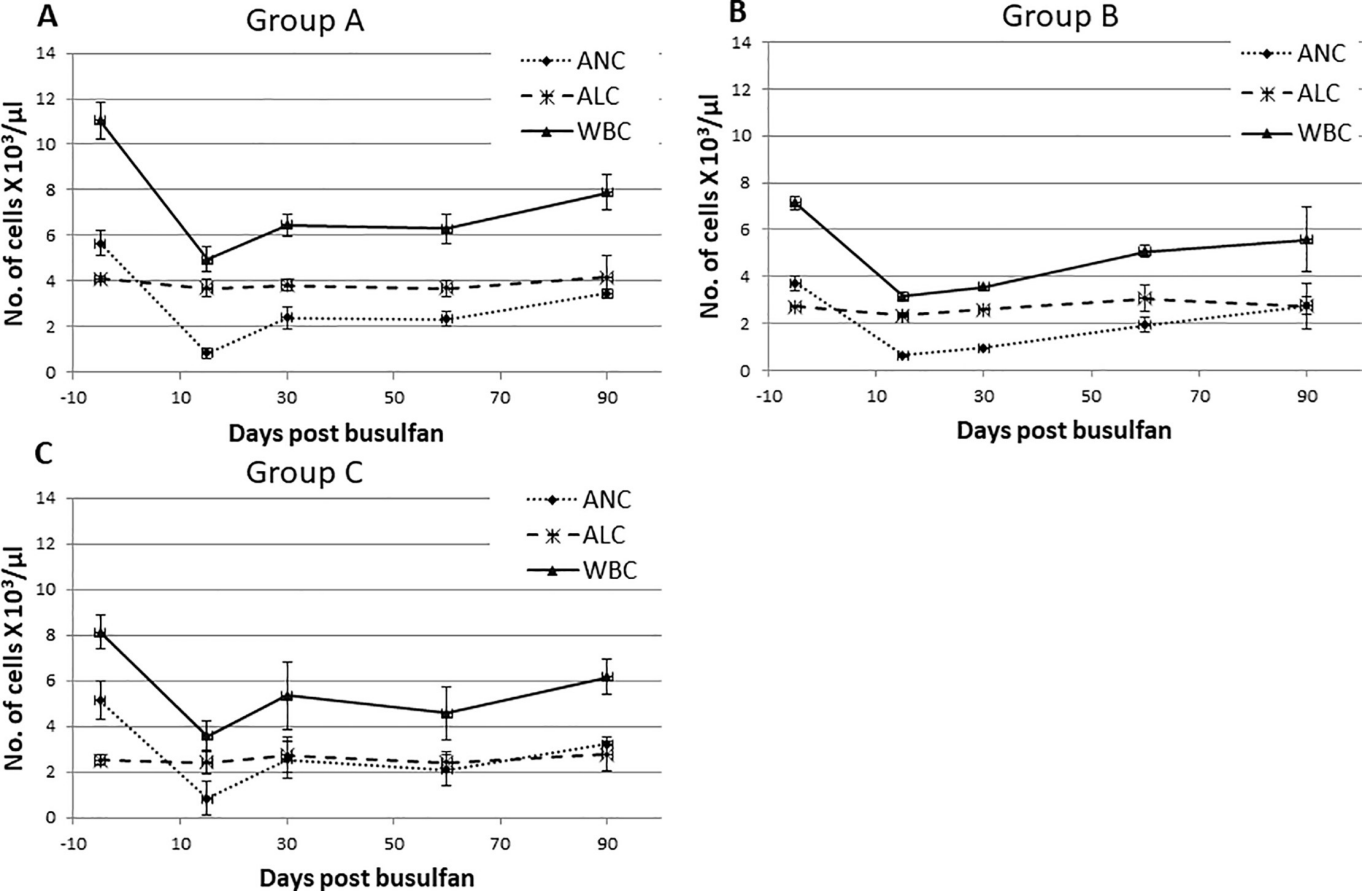
Peripheral blood differential counts post intravenous (IV) busulfan treatment is shown along automated white blood cell (WBC) counts. A. Peripheral blood absolute neutrophil count (ANC), absolute lymphocyte counts (ALC) and WBC count following 6.4mg/kg IV busulfan treatment (Group A). B. Peripheral blood ANC and WBC counts following 8 mg/kg IV busulfan treatment (Group B). C. Peripheral blood ANC, ALC and WBC counts following 9.6 mg/kg IV busulfan treatment (Group C).

**Fig 3:**
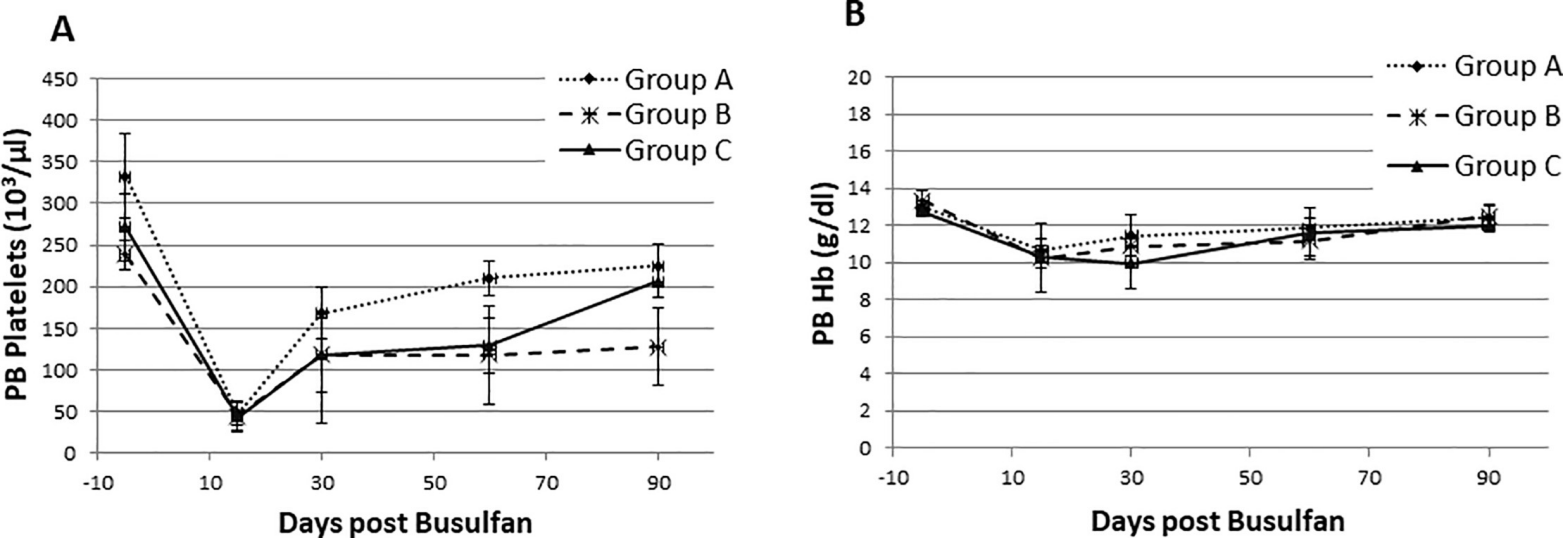
Effects of intravenous (IV) busulfan on peripheral blood (PB) parameters. **A.** Platelet counts in the PB are shown over the study period following busulfan treatment in all three groups of baboons. **B**. Hemoglobin (Hg) levels following busulfan treatment in all three groups of baboons is shown over the 90-day study period.

A busulfan test dose was performed to determine pharmacokinetics in one baboon. The area under the curve (AUC) was found to be 4820 uMol*min, which is very similar to what is observed in humans [13]. Increasing doses of IV busulfan at 6.4, 8.0, and 9.6 mg/kg were administered to three groups of animals (Fig 1) to assess the level of peripheral blood cytopenia caused by these treatments and the time to full hematologic recovery. The time to observe the greatest decrease of WBC, hemoglobin (Hb) and platelets compared to baseline values was 15 days after treatment in each group. As expected no significant effect was observed in the lymphocyte count at any dose tested. The absolute lymphocyte counts (ALC) following busulfan administration remained relatively unchanged (Fig 2) and busulfan is also known to be a non lymphotoxic agent. In addition, effects of busulfan administration on lymphocytes was analyzed by monitoring BM CD3+ T-lymphocyte counts by flow cytometry prior to and following therapy. The changes in BM CD3+ T-lymphocyte counts were not statistically significant (S1 Fig). Since there were no changes in absolute lymphocyte counts or CD3+ lymphocytes following busulfan therapy in any dose group other immune cell subsets (B-lymphocytes, Natural Killer cells) monitoring following busulfan administration was not part of the study. In contrast, at day 15 the absolute neutrophil count (ANC) in Group A, B and C decreased to: 0.84 ± 0.24, 0.63 ± 0.06 and 0.83 ± 0.14 x 10^3^/μL, respectively. (Fig 2A–2C). Compared to baseline levels, a significant decrease of ANC was observed in group A (85.78% ± 2.37%, *p* = 0.008), B (84.34% ± 1.25%, *p* = 0.06) and C (82.16% ± 5.24%, *p* = 0.047) (Fig 2B). Busulfan also caused thrombocytopenia, with average day 15 platelet numbers of 47 ± 13, 44 ± 17 and 43 ± 18 X10^3^/dl (Fig 3A), and anemia, with Hb levels of 10.6 ± 0.6, 10.2 ± 1.8 and 10.2 ± 0.6 g/dl, (Fig 3B) in groups A, B and C, respectively.

At day 90, the PB CBC results including WBC, Hb and platelets returned within normal ranges and were close to their pre-treatment values. Notably, the recovery of PB WBC counts at day 90 in the group receiving the highest dose of busulfan (Group C) displayed 76% ± 9% recovery (WBC count on Day 90, 6.14 ± 0.77 X 10^3^ /μl blood) while platelet count recovery in the same group at day 90 was 79.55% ± 13.64% (platelet count at day 90, 206 ± 18.56 X 10^3^/μl blood). In contrast, Group A and Group B had 68.4% ± 2.56% (Group A platelet count at day 90, 225.33 ± 25.67 x 10^3^/μl) and 53.9% ± 18.43% platelet recovery (Group B platelet count at day 90, 127.33 ± 46.28 X 10^3^/μl). WBC count recovery in Group A and Group B was 74.57% ± 11.36% (Group A WBC count 7.88 ± 0.76 x 10^3^/μl blood) and 64.67 ± 10.81% respectively (Group B WBC count 5.3 ± 0.68 X 10^3^/μl blood). Although lowest WBC and platelets counts were observed 15 days after busulfan treatment, the blood counts were maintained above threshold levels in all three groups of baboons. Yet failure to fully return PB counts to pre-treatment levels after 90 days of treatment may reflect possible changes in HSPC reserve affected particularly by the highest dose of busulfan administered (Group C). The lack of recovery of CFU was more pronounced in the group receiving the highest dose of busulfan despite relative normalization of PB counts, thus suggesting some level of replicative stress on stem/progenitor cells to maintain homoeostasis in PB counts.

Taken together, these results indicate that when using doses of IV busulfan between 6.4 and 9.6 mg/kg the regulatory systems governing hematopoietic homeostasis are likely to be maintained by reserved precursor and progenitor cells within the BM.

### Effects on bone marrow stem/progenitor cells

Although busulfan at 9 mg/kg body weight in combination with cyclophosphamide or total body irradiation has been shown as myeloablative in humans [17], data using busulfan as a single agent in the dose ranges tested in the current study is lacking. In order to understand effects of various doses of busulfan on the BM both prior to and following busulfan administration, BM biopsy samples were examined serially until day 90. Morphologic examination of BM biopsies revealed that at day 90 cellularity returned to 70–85% of pretreatment values for all three treatment groups (Table 2).

**Table 2:** Morphologic examination of Bone marrow biopsy specimens collected prior to and following busulfan treatment in baboon.

		Pre-Treatment		Post Busulfan Day 30		Post Busulfan Day 90	
		Cellularity (%)	Trilineage	Cellularity (%)	Trilineage	Cellularity (%)	Trilineage
**Group A**	**PA 7518**	20	Present	5	Decreased	5	Decreased
	**PA 7520**	80	Present	60	Present	60	Present
	**PA 7540**	85	Present	70	Present	85	Present
**Group B**	**PA 6537**	90	Present	30	Present	85	Present
	**PA 6683**	85	Present	50	Present	80	Present
	**PA 7519**	15	Present	5	Markedly Decreased	5	Markedly Decreased
**Group C**	**PA7527**	60	Present	50	Present	60	Present
	**PA7521**^**¶**^	40	Present	N/A	N/A	N/A	N/A
	**PA 7528**	NA	Present	60	Present	80	Present
	**PA 7533**	60	Present	30	Present	20	Present

^**¶**^**Animal** deceased 16 days’ post busulfan administration.

N/A: not available.

**Fig 4:**
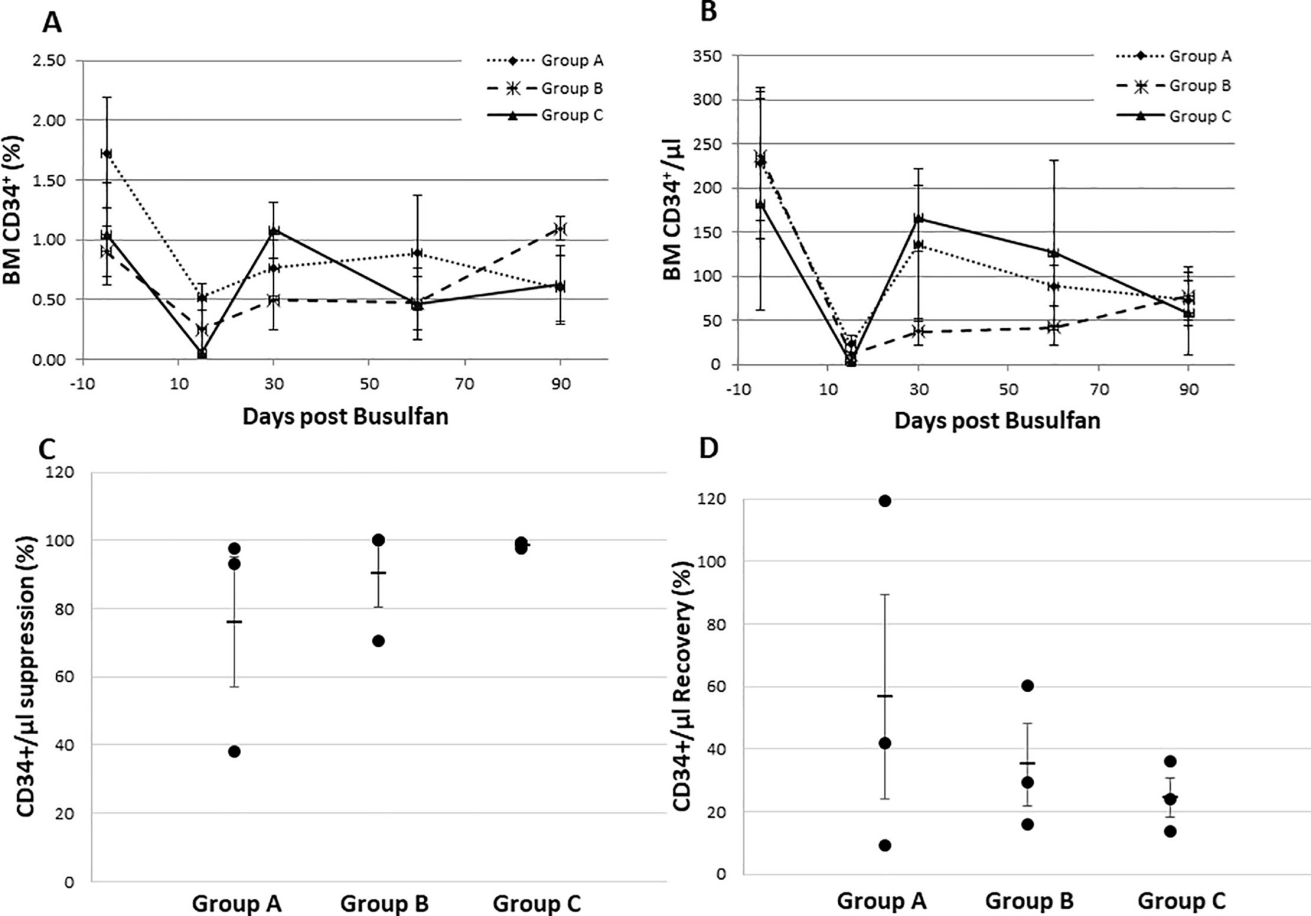
Bone marrow CD34+ cells kinetics following IV busulfan treatment in baboons. **A**. Frequency (%) of CD34+ cells in bone marrow following IV busulfan treatment over the course of the 90-day study period. **B**. Absolute number of bone marrow CD34+ cells following IV busulfan treatment for each group of baboons. **C**. Suppression of bone marrow CD34+ cell absolute values for each group of baboons 15-day post busulfan treatment relative to pretreatment levels are shown as percent values of mean ± SE in a bar graph. **D**. Recovery of absolute number of bone marrow CD34+ cells 90 after days of busulfan treatment also presented in a scatter graph using mean ± SE values of percent recovery relative to pretreatment levels.

To further examine the myelotoxicity of different doses of IV busulfan, we examined the CD34+ cell and CFU content of BM prior to and at different time points following busulfan therapy. As shown in Fig 4A, the proportion of marrow CD34+ cells detected in groups A, B and C at day 15 was 0.52 ± 0.11%, 0.25 ± 0.24% and 0.05 ± 0.03%, respectively (Group A vs Group C, *p* = 0.01; Group A vs Group B and Group B vs Group C, p-values were not statistically significant). Similarly, Fig 4B shows that the absolute number of BM CD34+ cells reached its nadir at day 15, with 23.17 ± 9.35 /μl, and 1.95 ± 0.98 /μl in groups A and C respectively, whereas 2 out of 3 baboons in Group B did not display any detectable CD34+ cells in the BM on day 15 after busulfan. Both the percentage and the absolute number of CD34+ cells varied depending on the dose of busulfan administered in each group of baboons, with Group C receiving the highest dose of busulfan displaying lowest number of CD34+ cells after busulfan administration. As shown in Fig 4C, the suppression of BM CD34+ cells relative to pretreatment levels reveals that Group A had 75.96% ± 19.12% (*p* = 0.16) suppression, while suppression was 90.13% ± 9.87% (*p* = 0.11) and 98.57% ± 0.60% (*p* = 0.27) in groups B and C, respectively. As shown in Fig 4D, the recovery of absolute number of BM CD34+ cells at day 90, compared to pre-treatment values, was 56.68% ± 32.73% (*p* = 0.25), 35.05% ± 13.05% (*p* = 0.16) and 24.40% ± 6.4% (*p* = 0.23) for groups A, B, and C, respectively.

**Fig 5:**
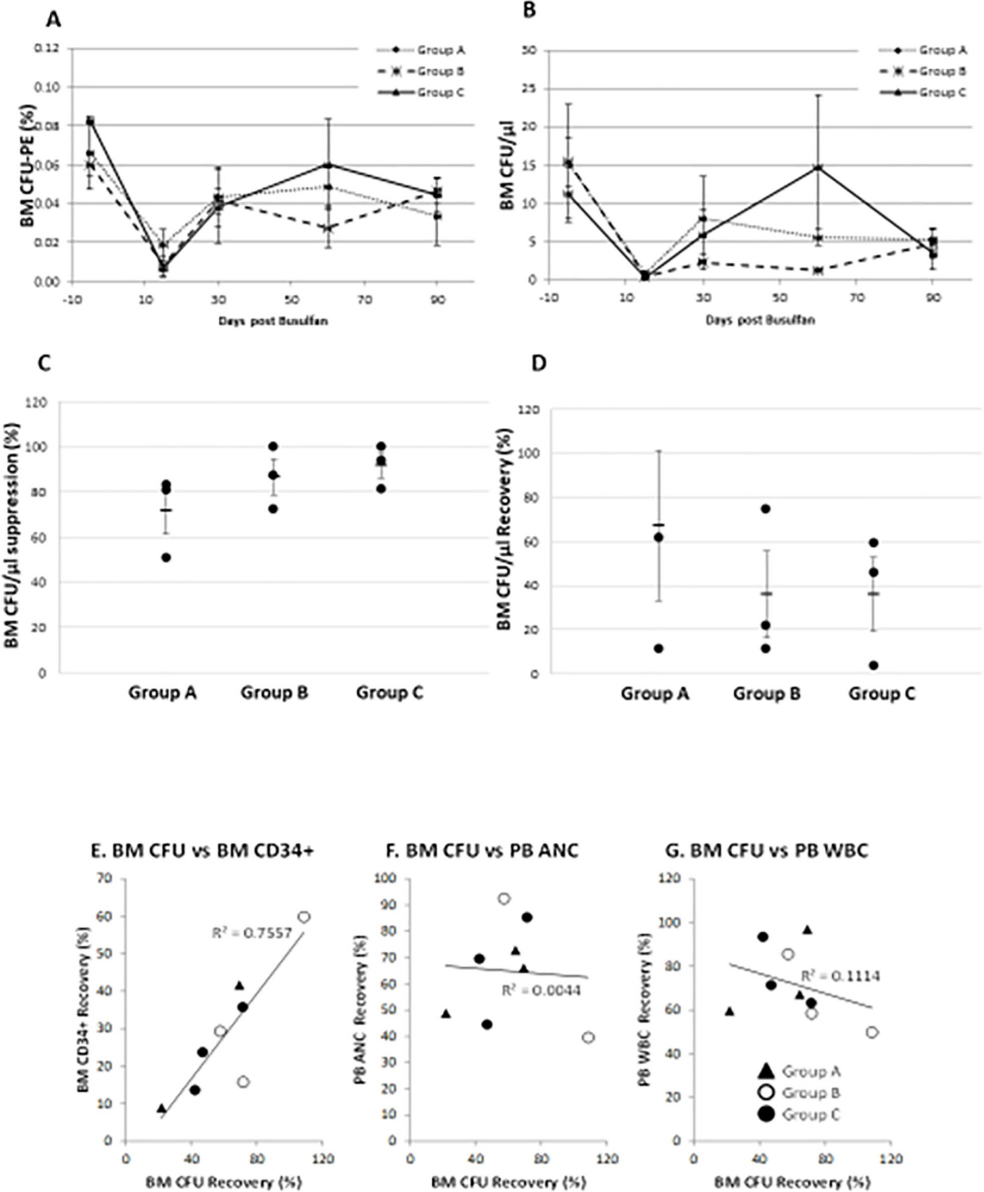
Alteration of the number of colony forming units (CFUs) in the bone marrow (BM) following intravenous (IV) busulfan treatment over the 90-day study period. **A**. The kinetics of frequency of CFUs in the BM following busulfan treatment depicted as plating efficiency (%) over the course of the 90-day study period. **B**. The kinetics of absolute number of BM CFUs following busulfan treatment for each group of baboons. **C**. Suppression of absolute number of CFUs in the BM using mean ± SE of percent suppression for each group of baboons 15-day post busulfan treatment to pretreatment levels. **D**. Recovery of CFUs in the BM 90 days after busulfan treatment relative to pretreatment levels are also shown using mean ± SE values of percent recovery relative to their pretreatment values. **E.** Correlation coefficient of bone marrow CFU recovery and CD34+ cell content recovery (excluding the outlier); **F.** Correlation coefficient of BM CFU recovery and recovery of absolute neutrophil count (ANC) in peripheral blood (PB) is shown; **G.** Correlation coefficient of BM CFU recovery and recovery of white blood cell (WBC) in peripheral blood (PB) is shown.

In order to examine the effect of busulfan in hematopoietic progenitor cell levels, we examined both CFU plating efficiency (Fig 5A) and absolute number of BM CFUs (Fig 5B) prior to and following busulfan administration in all three groups of baboons over the 90-day study period. As shown in Fig 5A, BM CFU at day 15 was 0.02% ± 0.008%, 0.01% ± 0.005% and 0.01 ± 0.004% in groups A, B and C, respectively. Suppression of BM CFU at day 15 in comparison to their pre-treatment value was 71.60% ± 10.37% (*p* = 0.107), 86.67% ± 7.99% (*p* = 0.011) and 91.68% ± 5.51% (*p* = 0.007) in groups A, B and C, respectively (Fig 5C). These findings indicate that on day 15 after treatment there was a more pronounced suppression of progenitors in Group B (*p* = 0.011) and Group C (*p* = 0.007). As shown in Fig 5D, recovery of absolute number of BM CFU at day 90 in groups A, B and C was 67.21% ± 33.92% (*p* = 0.35), 35.94% ±19.65% (*p* = 0.15) and 36.16% ± 16.76% (*p* = 0.19), respectively, compared to pre-treatment values. The recovery of CFU appeared to vary with the dose of busulfan administered, as the group which received the highest dose of busulfan experienced lowest recovery.

Data from one animal from Group A fell outside the range and was removed from analysis as an outlier. The correlation coefficient of CFU recovery and absolute CD34+ cell content recovery in the BM was studied and a correlation (R^2^ = 0.76, *p* = 0.028) between the two was observed (Fig 5E). The log transformation method was utilized to normalize the BM CD34 recovery to minimize the influence of the outlier data point from one animal. After transformation, in spite of the small sample size (n = 9) the correlation coefficient of log CD34 and CFU still remained marginally significant (*p* = 0.0541) with a R^2^ value of 0.4328, suggesting a possible correlation of BM CFU recovery with CD34+ cells recovery (S2 Fig). However, BM CFU recovery had no correlation with PB ANC (Fig 5F, R^2^ = 0.0040) or WBC recovery (R^2^ = 0.11) (Fig 5G). Notably, we observed that in animals receiving the highest dose of busulfan (Group C) although PB WBC, Hb and platelet values returned to normal levels, absolute number of BM CD34+ cells and CFU did not fully recover compared to pre-treatment levels (24.40% ± 6.4% and 36.15% ± 16.76%, respectively).

These observations suggest that only 25–35% of the BM hematopoietic stem/progenitor cell reserve is needed to sustain normal blood counts. Taken together, these data also suggest that there is a direct relationship between BM stem/progenitor cell suppression and busulfan dose, and an inverse relationship between BM stem/progenitor cell recovery and busulfan dose.

### Extra-hematopoietic toxicity

Baboons were assessed for extra-hematopoietic toxicity via weekly physical examinations performed by veterinary doctors and a toxicity chart was utilized for monitoring of study animals. Weekly physical examinations revealed no detectable physical signs of extra-hematopoietic organ impairment. All animals’ eating habits and daily excretions appeared to remain unaltered. As part of monitoring procedures, animals’ body weight was measured prior to busulfan treatment and weekly until day 90 and no significant weight loss was observed in the three groups. One baboon in the highest busulfan dose group died at day 16, likely due to septicemia. Although the animal did not display clinical signs/symptoms of septicemia, following necropsy bacterial thrombi were found in the liver, spleen and kidneys (data not shown). The animal was found dead in the cage. Then an additional animal was successfully added to Group C in order to have 3 animals fully treated and assessed in this group. No other animals displayed any signs of infection or septicemia.

## Discussion

Our current study in a steady state NHP model demonstrates that treatment with IV busulfan at a dose of 6.4 or 9.6 mg/kg causes reversible myeloablation with minimal to no evidence of severe extra-hematologic toxicity. It is notable that one of the animal in the highest busulfan dose group (9.6 mg/kg) died after 16 days of busulfan administration likely due to septicemia. We cannot exclude the fact that the death of the animal was a direct result of consequences of myeloablation resulting from busulfan administration.

These findings may support the utilization of IV busulfan in the treatment of myeloid malignancies relapsed or resistant to standard anthracycline-based chemotherapy. Busulfan is still considered the most effective chemotherapy drug in the conditioning regimen of patients undergoing HSCT for myeloid malignancies, especially using targeted IV doses [18,19]. However, it has never been tested in induction, or salvage therapies for the same malignancies. This study in a NHP model was aimed at providing preclinical evidence of effective but only transient myelosuppression induced by doses of IV busulfan lower than what is usually utilized in myeloablative HSCT. In order to demonstrate what dose of busulfan would have sufficient myelotoxicity without depleting irreversibly the HSC pool in the marrow, the toxic effect of this compound was tested *in vivo* by analyzing the hematopoietic recovery for 90 days following IV busulfan administration as well as the number and function of bone marrow CD34+ cells.

The survival of HSPC reserve in the BM can be severely compromised during exposure of alkylating agents [4,20–22] which can be monitored by evaluating CD34+ cell numbers in the BM [23–26]. In previous studies, we demonstrated that a sub-lethal dose of total body irradiation (250 cGy) in an NHP model resulted in profound suppression of absolute numbers of both CD34+ cells and CFUs in the BM, followed by a rebound exceeding the baseline numbers [27]. The recovery of CD34+ cells and CFU numbers in the BM following myelosuppression also correlate with normalization of PB WBC and platelet numbers [27]. However, in our current study the BM CD34+ cell and CFU content only recovered to about 25 to 35% of their pre-treatment levels by day 90 post-busulfan administration. Nevertheless, since PB WBC, Hb and platelet levels returned to normal values, we show that a 25 to 35% reserve of BM stem/progenitor cells are likely capable of sustaining normal hematopoiesis. Our results also indicate that while the recovery pattern of BM CD34+ cells and CFUs overlaps, it is distinct from the recovery pattern of relatively mature blood cells. Although the recovery of ANC did not vary based on dose of busulfan administered, the recovery of BM CD34+ cells and CFU both inversely correlated with busulfan dose as the lowest recovery was demonstrated in the group which received the highest dose of busulfan (Group C).

These results further validate that in a clinically relevant large animal model, IV busulfan targets the hierarchy of CD34+ cells and CFU rather than more mature functional blood cells, emphasizing that regulatory systems governing homeostasis are likely sustained by reserved BM stem/progenitor cells. Based on our findings of reversible myeloablation by busulfan up to 9 mg/kg body weight in a large animal model during physiological state and known similarities between normal and leukemic HSPCs [28], we speculate that IV busulfan could potentially target leukemic HSPCs including CFUs and CD34+ progenitor cells which will need to be further validated. Although the anti-leukemic effect of busulfan dose intensity remains controversial, busulfan dose intensity as a preparative regimen for patients with advanced myeloid malignancies undergoing allogeneic transplantation has shown better outcomes [29]. Hematologic responses to busulfan either given via the oral or IVroutes in large animals is not available. In an earlier study busulfan with a single oral dose (100 mg/m2) without stem cell rescue has been attempted in leukemia patients [30]. It is well known that bioavailability of busulfan following oral administration is less predictable than intravenous busulfan. Furthermore, it is conceivable that IV busulfan can be utilized in chemotherapy regimens for hematologic myeloid malignancies, possibly in combination with other standard agents such as anthracyclins or cytarabine. Unfortunately, a large animal model for leukemia does not exist, and therefore pilot studies in human patients will be needed to determine whether the busulfan doses tested in this study will have a measurable anti-leukemic effect.

Our findings in NHP model will allow us to design a phase 1/2 clinical study where IV busulfan can be administered alone, or in combination with a standard purine analogue, in patients with AML relapsed/refractory to conventional chemotherapy.

## Supporting information

S1 Checklist ARRIVE Checklist 2014 Mahmud et al August 2018. (PDF)

S1 Fig Bone marrow CD3+ T lymphocyte count following busulfan administration. Bone marrow CD3+ lymphocytes was measured prior to and following busulfan administration in all three groups of baboons (Group A, Group B and Group C). The data (mean ± SEM) is expressed as absolute number of CD3+ cells per microliter bone marrow. The CD3+ T cell number pre and post busulfan administration was not statistically significant, paired student t test *p* <0.05).
(TIF)

S2 Fig Correlation coefficient of bone marrow CFU and CD34+ cell recovery following busulfan administration in baboon. Bone marrow CFU and log transformed CD34+ cell content recovery including all 9 baboons.
(TIF)
