# Supplementary figures and images for: Preclinical IV busulfan dose-finding study to induce reversible myeloablation in a non-human primate model
Source: PLoS One. 2018 Nov 29;13(11):e0206980. doi: 10.1371/journal.pone.0206980 (PMC6264479; doi:10.1371/journal.pone.0206980)

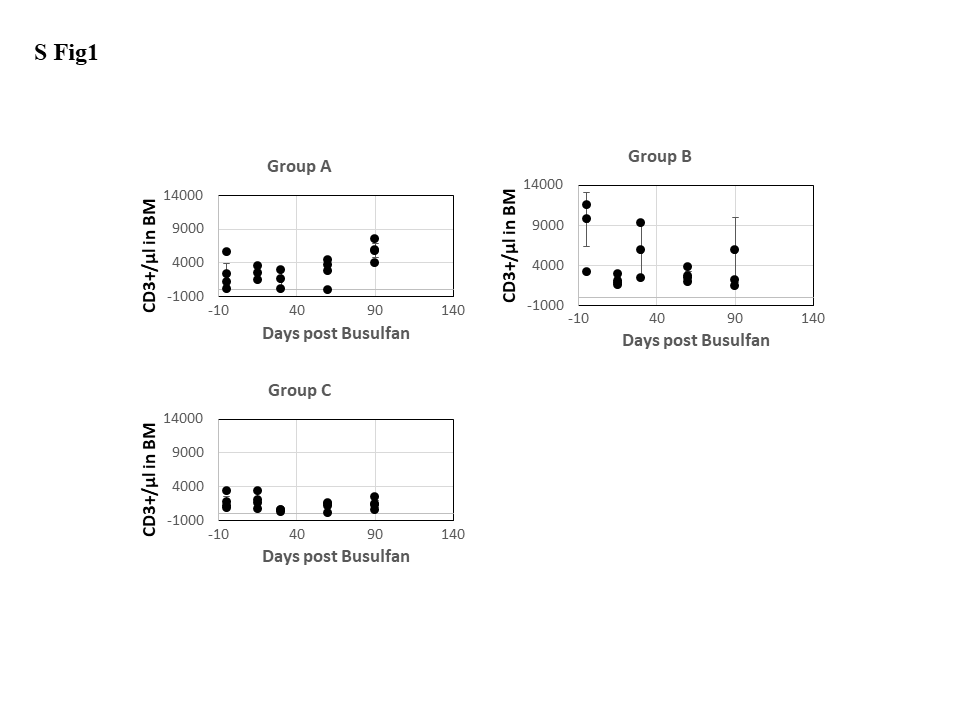

Supplement: S1 Fig — Bone marrow CD3+ lymphocytes was measured prior to and following busulfan administration in all three groups of baboons (Group A, Group B and Group C). The data (mean ± SEM) is expressed as absolute number of CD3+ cells per microliter bone marrow. The CD3+ T cell number pre and post busulfan administration was not statistically significant, paired student t test p <0.05). (TIF) [file pone.0206980.s002.tif]

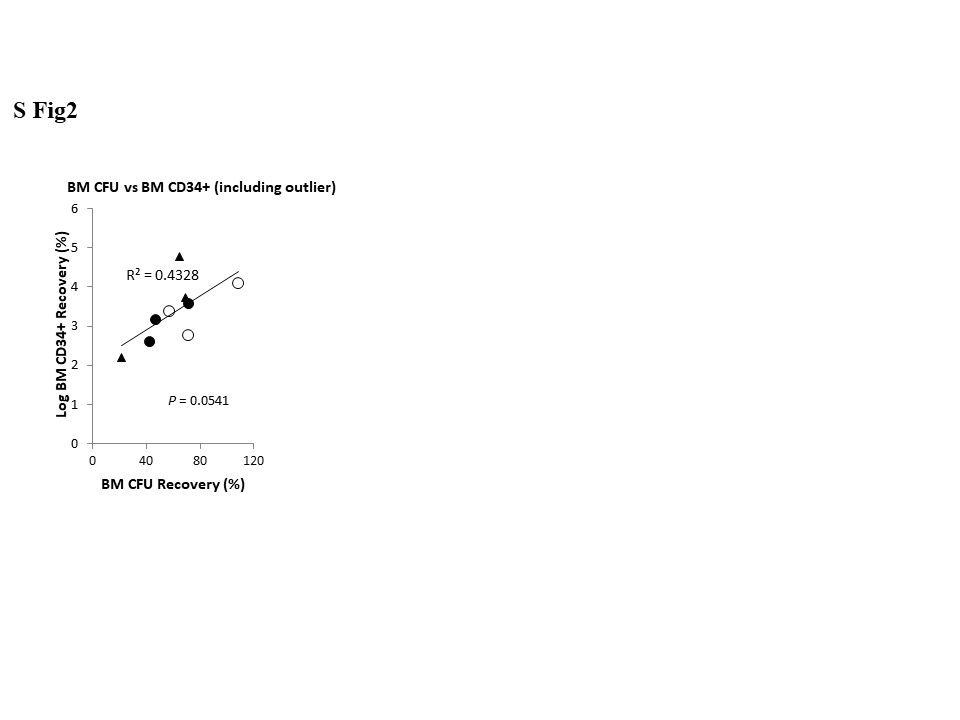

Supplement: S2 Fig — Bone marrow CFU and log transformed CD34+ cell content recovery including all 9 baboons. (TIF) [file pone.0206980.s003.tif]
